# Major Histocompatibility Complex Class I-Related Chain A (MICA) Allelic Variants Associate With Susceptibility and Prognosis of Gastric Cancer

**DOI:** 10.3389/fimmu.2021.645528

**Published:** 2021-03-31

**Authors:** Karen Toledo-Stuardo, Carolina H. Ribeiro, Andrea Canals, Marcela Morales, Valentina Gárate, Jose Rodríguez-Siza, Samantha Tello, Marco Bustamante, Ricardo Armisen, Douglas J. Matthies, Gerald Zapata-Torres, Patricio González-Hormazabal, María Carmen Molina

**Affiliations:** ^1^Immunology Program, Faculty of Medicine, Institute of Biomedical Sciences (ICBM), University of Chile, Santiago, Chile; ^2^Biostatistics Program, School of Public Health, University of Chile, Santiago, Chile; ^3^Academic Direction, Clínica Santa María, Santiago, Chile; ^4^Department of Surgery (Oriente), Hospital del Salvador, University of Chile, Santiago, Chile; ^5^Center of Genetics and Genomics, Faculty of Medicine Clínica Alemana, Institute for Sciences and Innovations in Medicine (ICIM), Universidad del Desarrollo, Santiago, Chile; ^6^Department of Inorganic and Analytical Chemistry, Faculty of Chemical and Pharmaceutical Sciences, University of Chile, Santiago, Chile; ^7^Human Genetics Program, Institute of Biomedical Sciences (ICBM), University of Chile, Santiago, Chile

**Keywords:** gastric cancer, MICA gene, MICA polymorphism, MICA-129, MICA alleles

## Abstract

Gastric cancer (GC) is the fifth most prevalent type of cancer worldwide. Gastric tumor cells express MICA protein, a ligand to NKG2D receptor that triggers natural killer (NK) cells effector functions for early tumor elimination. *MICA* gene is highly polymorphic, thus originating alleles that encode protein variants with a controversial role in cancer. The main goal of this work was to study *MICA* gene polymorphisms and their relationship with the susceptibility and prognosis of GC. Fifty patients with GC and 50 healthy volunteers were included in this study. MICA alleles were identified using Sanger sequencing methods. The analysis of *MICA* gene sequence revealed 13 MICA sequences and 5 MICA-short tandem repeats (STR) alleles in the studied cohorts We identified MICA^*^002 (^*^A9) as the most frequent allele in both, patients and controls, followed by MICA^*^008 allele (^*^A5.1). MICA^*^009/049 allele was significantly associated with increased risk of GC (OR: 5.11 [95% CI: 1.39–18.74], *p* = 0.014). The analysis of MICA-STR alleles revealed a higher frequency of MICA^*^A5 in healthy individuals than GC patients (OR = 0.34 [95% CI: 0.12–0.98], *p* = 0.046). Survival analysis after gastrectomy showed that patients with MICA^*^002/002 or MICA^*^002/004 alleles had significantly higher survival rates than those patients bearing MICA^*^002/008 (*p* = 0.014) or MICA^*^002/009 (MICA^*^002/049) alleles (*p* = 0.040). The presence of threonine in the position MICA-181 (MICA^*^009/049 allele) was more frequent in GC patients than controls (*p* = 0.023). Molecular analysis of MICA-181 showed that the presence of threonine provides greater mobility to the protein than arginine in the same position (MICA^*^004), which could explain, at least in part, some immune evasion mechanisms developed by the tumor. In conclusion, our findings suggest that the study of MICA alleles is crucial to search for new therapeutic approaches and may be useful for the evaluation of risk and prognosis of GC and personalized therapy.

## Introduction

Gastric cancer (GC) is the fifth most common neoplasm and the third leading cause of cancer-related death worldwide (1), accounting for 783,000 deaths in 2018 according to GLOBOCAN data.

The absence of early clinical tools for GC diagnosis decreases the success of therapies and survival of patients, which raises the question of the necessity to explore novel biological biomarkers of the disease (2–4). In this context, the major histocompatibility complex class I-related protein A (MICA) may provide relevant information about the pathological changes in the gastrointestinal mucosa. The healthy gastric mucosa express low levels of this protein, while the tumor tissue overexpresses it (5–7). MICA expression in the carcinogenesis process is regulated by transcriptional, translational and/or post-translational modification mechanisms (8, 9) that could be associated with *Helicobacter pylori* or Epstein Barr-virus infection (10, 11).

MICA is a ligand to natural killer group 2D (NKG2D), an activating receptor (12) that is important for the anti-tumor immune response (13). NKG2D is expressed by cytotoxic lymphocytes, including natural killer (NK) cells, γδ T cells, and CD8^+^ T cells. NK cells constitute the first line of defense against intracellular pathogens; they also contribute to the maintenance of mucosal homeostasis and the development of efficient immune responses against cancer (14). NKG2D binding to MICA on target cells triggers NK cell cytolytic activation, which results in target cell lysis through the release of granzyme and perforin by the effector cell (15). However, tumors have developed several strategies to evade the immune response, such as the proteolytic shedding of MICA from the cell membrane (13). The resulting soluble form of MICA induces the downregulation of NKG2D receptor on NK cells, compromising the immune response mediated by these and other cytolytic cells (16).

The human *MICA* gene is highly polymorphic; indeed, it has been reported more than 110 MICA alleles that encode over 100 protein variants (http://hla.alleles.org/data/mica.html). The human *MICA* gene is located in chromosome 6p21.3, 46 kb distant from HLA-B, and consists of six exons: exon 1–6 encode the leader peptide, the three extracellular protein domains (α1, α2, and α3), the transmembrane region, and the hydrophobic cytoplasmatic tail, respectively (17). Exon 5 contains a short tandem repeat (STR) with a variable number of GCT triplet repeats, which encode alanine (Ala). A second nomenclature for MICA alleles, MICA-STR (18), has thus emerged based on the presence of these repetitions; for instance, MICA^*^A5 consists of five repetitions of GCT. Such STR affects the length of the transmembrane region, as in the case of MICA^*^A9, which contains nine GCT repetitions, which consequently codifies nine residues of alanine (18). Interestingly, the MICA^*^A5.1 (prototype MICA^*^008) variant produces an insertion of guanine at position 952 (Chr6: 31380161-31380162 on Assembly GRCh37) in the transmembrane region, changing the reading frame to a pre-mature stop codon and generating a protein with a GPI anchor, which results in the recruitment of MICA to exosomes and release of the protein in the form of extracellular vesicles (EVs) (19, 20).

MICA polymorphisms have been previously studied in cancer, especially the MICA-129 residue, which is associated to the presence of methionine (Met) or valine (Val), where MICA-129 Met has shown a strong interaction with NKG2D, leading to the downregulation of the receptor more efficiently than MICA-129 Val (21). On the other hand, MICA-129 Val has been associated to an increase in soluble MICA (sMICA) levels in multiple myeloma, which induces NKG2D downregulation and contributes to immune evasion (16). Furthermore, several MICA transmembrane regions, based on the GCT repetitions, have been associated to higher susceptibility to certain types of cancers. For instance, MICA^*^A9 allele has been proposed to confer a risk for GC (22), while MICA^*^A5.1 allele may be associated with increased susceptibility to oral squamous cell carcinoma in Japanese patients (23). Since MICA alleles vary among human populations and generate proteins with different biological properties that may result in variable disease susceptibility, we decided to study MICA gene polymorphisms and susceptibility to gastric cancer and their relationship with the tumor progression.

## Materials and Methods

### Patients and Healthy Controls Samples

#### Gastric Cancer Patients Tissue Samples

During June 2011 and August 2015, a total of 50 patients (17 female, 33 male) aged 65.4 ± 10.3 years (range, 49–78 years) with confirmed diagnosis of gastric adenocarcinoma and treated at the Department of Gastrointestinal Surgery, Hospital del Salvador (Santiago, Chile), were enrolled in this study. Fresh primary gastric tumor tissue samples were obtained at the time of surgery and immediately processed for genomic DNA extraction.

#### Healthy Controls Blood Samples

Blood samples were obtained from 50 healthy donors (24 female, 26 male) aged 47.3 ± 15.6 years (range, 30–61 years) without prior gastrointestinal diseases or any type of cancer, who lived in Santiago, Chile. Blood samples were collected in 4 mL EDTA-containing vacutainer tubes (BD Biosciences, San José, CA, USA) for genomic DNA extraction.

#### Ethical Considerations

This study was approved by the Committee on Human Ethics Investigation of the Faculty of Medicine, University of Chile, and the Committee on Scientific Ethics of the Metropolitan Health Service of the Chilean Government. All patients and healthy volunteers signed a written Informed Consent for tissue and blood donation. Name and identification of patients and controls were omitted and rendered anonymous before data analysis.

### Clinicopathological Data

The clinicopathological information included patient gender, age, primary tumor location, tumor size, Lauren's histological classification, Bormann classification (24), lymph node metastasis and tumor stage according to the American Joint Committee on Cancer, AJCC, 7th edition (25). Histopathological analysis was carried out by the team of pathologists from Hospital del Salvador (Santiago, Chile). The histological differentiation grade was based on the World Health Organization (WHO) classification (26). Patient survival was assessed for 36 months after surgery or until death due to tumor-specific disease.

### Extraction and Purification of Genomic DNA

The genomic DNA was obtained from ~30 mg of tumor tissue using the E.Z.N.A Tissue DNA kit (Omega, Bio-tek, USA), following the manufacturer's instructions. The genomic DNA from blood samples was isolated using the salting out method previously described by Subbarayan PR and collaborators (27).

### Measurement of DNA Purity and Integrity

The genomic DNA purity was assessed according to the mean 260/280 nm ratio in a Synergy™ microplate reader (Biotek, Winooski, VT, USA). The genomic DNA integrity was verified by electrophoresis using a 1% agarose gel (40 mg of analytical grade Agarose, 40 mL of Tris-borate-EDTA buffer) with 0.5 μL of ethidium bromide solution (Sigma-Aldrich/Merck KGaA, Darmstadt, Germany). The DNA bands were visualized using a UV transilluminator.

### MICA Genotyping

To amplify the *MICA* gene of GC patients and healthy volunteers by PCR, we used two pairs of specific primers to 2-3 and 4-5 exons of the *MICA* gene, and MICA genotyping was carried out using bidirectional Sanger sequencing methods. Forward primer (2-3 exons): 5′ TGAAATCCTCGTTCTTGTCCCTTTGC 3′, Reverse primer (2-3 exons): 5′ AGGGTCCTCTACTTGCCCTGATTAC 3′; Forward primer (4-5 exons): 5′ TCAGCCAGAGTGAGAACAGTGAAGA 3′, Reverse primer (4-5 exons): 5′ TCATCCCCTGTTATGGAAGCCTTGTC 3′. The PCR reactions were analyzed by 0.8% agarose gel electrophoresis. The amplicons were purified using the Wizard SV Gel and PCR Clean-Up System Purification Kit (Promega, USA). The amplified products were sequenced on an ABI PRISM-3500 XL sequencer (Applied Biosystems, Foster City, CA). The genomic sequences of each sample were analyzed using Chromas 2.4 Viewer and were manually verified. These sequences were then compared with *MICA* allelic genotype sequences obtained from the IPD-IMGT/HLA database (28) (http://hla.alleles.org/data/mica.html). MICA allelic genotypes were obtained according to reference sequences of specific MICA alleles. The presence of heterozygous alleles in the sequences was assigned by Chromas and verified in the electropherogram. The exon 5 was carefully analyzed, manually, due to the presence of STR or microsatellites with different lengths, especially in heterozygous patients, as Sanger sequencing of this exon frequently results in base overlap. To validate our methodology, we analyzed two available cell lines with known MICA alleles, which included AGS ATCC CRL-1739 human gastric cancer cell line (which is homozygous for MICA^*^010) (29) and PC-3 ATCC CRL-1435 human prostate cancer cell line (which is heterozygous for MICA^*^008 and ^*^012) (30). MICA^*^009 and MICA^*^049 alleles differ in one amino acid in the exon 6. Since the differential residue was not distinguished in this study, this allele was named as MICA^*^009/049; accordingly, allele MICA^*^002/009 was also named MICA^*^002/049.

### Molecular Analysis

MICA protein sequence and its isoforms were retrieved from UniProtKB1 (31) (https://www.uniprot.org) in its FASTA format. A protein-protein BLAST (32) (http://blast.ncbi.nlm.nih.gov) was further performed to search for a protein template with the highest sequence identity to MICA, which resulted in PDB code 1HYR (17) with a 2.7 Å resolution. A sequence alignment with this template was then carried out using Clustal Omega server (33) (https://www.ebi.ac.uk). This target sequence alignment was fed on the Swiss-Model server (34) (https://swissmodel.expasy.org) to build the models of the proteins, which were then solvated *in silico* with water using a rectangular shape and neutralized with ions to a concentration of 0.15 M of NaCl to mimic physiological environment using the CHARMM-GUI server (35). Periodic boundary conditions were applied, and a minimization procedure was performed using a maximum number of 5,000 steps of conjugated gradient followed by 2,500 steps of steepest descent run. Next, the NVT ensemble was used for subsequent equilibration steps at 303.15 K using a timestep of 0.001 ps for 125 ps. NPT dynamic runs were used for production runs with a timestep of 0.004 ps for a period of 500 ns. The simulations were carried out using Amber14 (36) suite of programs.

### Statistical Analysis

Descriptive analysis of demographic data and clinicopathological characteristics of cases and controls were performed using median and interquartile range for quantitative variables and absolute and percentage frequency distributions for categorical variables.

The distribution of MICA-sequence alleles and MICA-STR alleles among cases and controls, and the proportion of each allele among cases and controls were compared using Fisher's Exact Test. In addition, logistic regressions were adjusted for the prediction of GC by comparing each allele with the others, obtaining the odds ratio (OR) with their respective confidence intervals (95%, CI).

The distribution of genotypes of each MICA residue between cases and controls was also compared using Fisher's Exact Test.

Survival curves of GC patients, according to the presence of MICA alleles, were obtained using the Kaplan-Meier method and compared using the Log-rank test.

Statistical analysis was performed using Stata 14 software, and a *p*-value < 0.05 was considered significant.

## Results

### Clinicopathological Characteristics of GC Patients

Patients demographic characteristics and clinicopathological features of tumors are described in Table 1. Tumor size was given as the maximum tumor diameter measured on the freshly resected stomach. Most of GC patients (64%) presented with a tumor size higher than 5 cm. In 74.5% of patients, the tumors had cardia location. According to Lauren's histological classification, 27 out of 50 patients had intestinal type GC, 13 had diffuse type GC, and 10 patients presented with mixed type GC. Borrmann's classification showed that the gastric tumors were mainly at stage III (46%), followed by stage IV (11%), which is related to the TNM staging classification, where 41 patients were found at stage III-IV. Additionally, 46% of the patients showed *Helicobacter pylori* infection.

Table 1Demographic data and clinicopathological characteristics of gastric cancer patients and controls.

**Table:** 

**Demographic data**	**Controls** ***n*** **=** **50**		**Gastric patients** ***n*** **=** **50**	
	**Median/n**	**IQR/%**	**Median/n**	**IQR/%**
Age	49	(30-61)	67	(49-78)
Gender				
Female	24	(48.0%)	17	(34.0%)
Male	26	(52.0%)	33	(66.0%)

**Table:** 

**Clinicopathological characteristics**		**n**	**%**
Location of tumor	Cardia	10	(21.3%)
	No cardia	35	(74.5%)
	Both	2	(4.3%)
Tumor size, cm	≤5	18	(36.0%)
	>5	32	(64.0%)
Lauren's classification	Intestinal type	27	(54.0%)
	Diffuse type	13	(26.0%)
	Mixed type	10	(20.0%)
Borrmann's classification	I (Polypoid/fungating)	2	(4.0%)
	II (Superficial spreading)	3	(6.0%)
	III (Ulcerating)	23	(46.0%)
	IV (Linitis plastica)	11	(22.0%)
	V (Unclassified)	5	(10.0%)
	Not documented	6	(12.0%)
Peritoneal cytology	Positive	5	(10.0%)
	Negative	39	(78.0%)
	Not documented	6	(12.0%)
TNM staging	I, II	8	(16.3%)
	III, IV	41	(83.7%)
*Helicobacter pylori*	Positive	23	(46.0%)
	Negative	27	(54.0%)

*IQR, Interquartile range*.

### MICA Allelic Frequency in GC Patients and Healthy Individuals

The analysis of *MICA* gene allowed us to identify 13 MICA-sequence alleles and 5 MICA-STR alleles in the studied population (Table 2). The distribution of MICA allelic frequencies identified in this study was different between GC patients and healthy volunteers (*p* = 0.024). We observed that the most frequent MICA allele found in both, patients and controls was the MICA^*^002 (^*^A9) allele, which was followed by the MICA^*^008 (^*^A5.1) allele. Together, both alleles represented more than 50 percent of all the alleles identified in our analysis. As shown in Supplementary Table 1, the MICA genotype frequency distribution in GC patients and healthy individuals did not show significant differences. The MICA^*^002/008 (^*^A9/A5.1) heterozygous genotype was the most common in both groups, representing 18.2 and 20% in GC patients and healthy controls, respectively.

**Table 2:** MICA allelic frequency in gastric cancer patients and healthy controls.

**MICA allele**	**GC patients**		**Healthy controls**		OR^*^	**95% CI**	***p*-value**
	**Number 2n = 88**	**(%)**	**Number 2n = 100**	**(%)**			
**MICA-sequence**							
MICA^*^001	3	(3.4%)	0	(0.0%)	–	–	
MICA^*^002	32	(36.4%)	32	(32.0%)	1.21	(0.66–2.22)	0.529
MICA^*^004	7	(8.0%)	17	(17.0%)	0.42	(0.17–1.07)	0.070
MICA^*^007	2	(2.3%)	3	(3.0%)	0.75	(0.12–4.61)	0.758
MICA^*^008	23	(26.1%)	21	(21.0%)	1.33	(0.68–2.62)	0.407
MICA^*^009/049	12	(13.6%)	3	(3.0%)	5.11	(1.39–18.74)	**0.014**
MICA^*^010	4	(4.6%)	7	(7.0%)	0.63	(0.18–2.24)	0.478
MICA^*^011	2	(2.3%)	5	(5.0%)	0.44	(0.08–2.34)	0.336
MICA^*^012	0	(0.0%)	2	(2.0%)	–	–	–
MICA^*^017	2	(2.3%)	2	(2.0%)	1.14	(0.16–8.26)	0.897
MICA^*^019	1	(1.1%)	6	(6.0%)	0.18	(0.02–1.52)	0.116
MICA^*^027	0	(0.0%)	1	(1.0%)	–	–	–
MICA^*^099	0	(0.0%)	1	(1.0%)	–	–	–
**MICA-STR**							
MICA^*^A4	5	(5.7%)	5	(5.0%)	1.14	(0.32–4.09)	0.835
MICA^*^A5	5	(5.7%)	15	(15.0%)	0.34	(0.12–0.98)	**0.046**
MICA^*^A5.1	23	(26.1%)	21	(21.0%)	1.33	(0.68–2.62)	0.407
MICA^*^A6	20	(22.7%)	25	(25.0%)	0.88	(0.45–1.73)	0.716
MICA^*^A9	33	(37.5%)	32	(32.0%)	1.28	(0.70–2.33)	0.429
MICA-NA	2	(2.3%)	2	(2.0%)	–	–	–

* *Odds ratio (OR) of gastric cancer for each allele compared to the rest of alleles. The table included 44 of 50 patients whose alleles have been identified*.

*NA, Not applicable; it corresponds to an allele without single tandem repetitions. Bold values indicate a statistically significant difference with a p-value < 0.05*.

We found that the MICA^*^009/049 allele frequency was significantly higher in GC patients than in healthy controls (*p* = 0.007), with a OR: 5.11 (95% CI: 1.39–18.74, *p* = 0.014). Additionally, we detected a higher frequency of MICA^*^A5 allele in healthy individuals than in GC patients, with a OR = 0.34 [95% CI: 0.12–0.98, *p* = 0.046) (Table 2). These results indicate that the presence of the MICA^*^A5 allele may be a protective factor in this type of tumor.

### MICA Polymorphisms and Development of GC

We next analyzed the genotypes of the main amino-acids of MICA ectodomain in GC patients and healthy controls (Table 3). The results showed that the only amino-acid that varies between patients and controls was the residue in position 181 (*p* = 0.023), which corresponds to the substitution of threonine by arginine. This change defines the difference between MICA^*^004 allele (arginine) and MICA^*^009/049 allele (threonine). We observed that the majority of GC patients had a Thr/Thr genotype (42/50), while the Arg/Arg genotype was not identified in these patients. Patients with a Thr/Thr or Thr/Arg genotype did not show a significant difference in the tumor size or differentiation grade.

**Table 3:** MICA amino-acids genotypes of GC patients and healthy controls.

**MICA-amino acid**	**SNP**	**Genotype**	**GC patients (*****n*** **=** **50)**		**Healthy controls (*****n*** **=** **50)**		*p*-value^*^
			**n**	**(%)**	**n**	**(%)**	
MICA-6	rsrs9380254	Pro/Pro	0	(0.0%)	1	(2.0%)	0.525
		Arg/Pro	4	(8.0%)	6	(12.0%)	
		Arg/Arg	46	(92.0%)	43	(86.0%)	
MICA-14	rs1063630	Gly/Gly	7	(14.0%)	5	(10.0%)	0.579
		Gly/Trp	23	(46.0%)	29	(58.0%)	
		Trp/Trp	20	(40.0%)	16	(32.0%)	
MICA-24	rs1051785	Ala/Ala	44	(88.0%)	48	(96.0%)	0.134
		Ala/Thr	6	(12.0%)	2	(4.0%)	
MICA-36	rs1051786	Cys/Cys	10	(20.0%)	7	(14.0%)	0.540
		Tyr/Cys	25	(50.0%)	30	(60.0%)	
		Tyr/Tyr	15	(30.0%)	13	(26.0%)	
MICA-91	rs41558312	Gln/Gln	48	(96.0%)	48	(96.0%)	0.691
		Gln/Arg	2	(4.0%)	2	(4.0%)	
MICA-122	rs1051790	Leu/Leu	31	(62.0%)	31	(62.0%)	1.000
		Leu/Val	17	(34.0%)	18	(36.0%)	
		Val/Val	2	(4.0%)	1	(2.0%)	
MICA-125	rs1051791	Glu/Glu	47	(94.0%)	50	(100.0%)	0.121
		Glu/Lys	3	(6.0%)	0	(0.0%)	
MICA-129	rs1051792	Met/Met	10	(20.0%)	7	(14.0%)	0.680
		Met/Val	26	(52.0%)	30	(60.0%)	
		Val/Val	14	(28.0%)	13	(26.0%)	
MICA-151	rs41560824	Met/Met	48	(96.0%)	45	(90.0%)	0.218
		Met/Val	2	(4.0%)	5	(10.0%)	
MICA-156	rs3819268	His/His	48	(96.0%)	48	(96.0%)	0.691
		His/Leu	2	(4.0%)	2	(4.0%)	
MICA-173	rs1051794	Glu/Glu	15	(30.0%)	13	(26.0%)	0.758
		Glu/Lys	26	(52.0%)	30	(60.0%)	
		Lys/Lys	9	(18.0%)	7	(14.0%)	
MICA-175	rs1131896	Gly/Gly	26	(52.0%)	21	(42.0%)	1.667
		Gly/Ser	21	(42.0%)	25	(50.0%)	
		Ser/Ser	3	(6.0%)	4	(8.0%)	
MICA-181	rs1131897	Arg/Arg	0	(0.0%)	1	(2.0%)	**0.023**
		Thr/Arg	8	(16.0%)	18	(36.0%)	
		Thr/Thr	42	(84.0%)	31	(62.0%)	
MICA-206	rs1131898	Gly/Gly	9	(20.5%)	7	(14.0%)	0.662
		Gly/Ser	23	(52.3%)	30	(60.0%)	
		Ser/Ser	12	(27.3%)	13	(26.0%)	
MICA-210	rs1051798	Arg/Arg	12	(27.3%)	13	(26.0%)	0.290
		Arg/Trp	23	(52.3%)	30	(60.0%)	
		Trp/Trp	9	(20.5%)	7	(14.0%)	
MICA-213	rs1140700	Ile/Ile	4	(9.1%)	3	(6.0%)	0.291
		Thr/Ile	20	(45.5%)	31	(62.0%)	
		Thr/Thr	20	(45.5%)	16	(33.0%)	
MICA-215	rs1051799	Ser/Ser	9	(20.5%)	7	(14.0%)	0.662
		Ser/Thr	23	(52.3%)	30	(60.0%)	
		Thr/Thr	12	(27.3%)	13	(26.0%)	
MICA-251	rs1063635	Gln/Gln	23	(46.0%)	16	(32.0%)	0.288
		Gln/Arg	23	(46.0%)	31	(62.0%)	
		Arg/Arg	4	(8.0%)	3	(6.0%)	

* *Fisher's exact test. Bold values indicate a statistically significant difference with a p-value < 0.05*.

Additionally, we evaluated the possible association between clinical features related to the risk of developing GC and the MICA-129 polymorphism, since in position 129 resides one of the most studied residues that determine a variable affinity to NKG2D receptor. We found that MICA-129 single nucleotide polymorphism (SNP) (rs1051792) showed a strong linkage disequilibrium with other SNPs related to residues 36, 173, 206, 210, and 215. We did not identify associations between these genotypes and tumor size or differentiation grade. The presence of positive peritoneal cytology was identified in only five patients and, for this reason, it was not possible to classify the patients into groups according to this pathological characteristic, although positive peritoneal cytology was detected only in patients with Met/Val and Val/Val MICA-129 genotype (data no shown).

We studied the relevance of the MICA alleles, both MICA-sequence and MICA-STR, on the clinicopathological characteristics of the tumor. We observed that the majority of patients with the MICA^*^A9/A6 heterozygote allele showed mainly a poorly differentiated tumor (*p* = 0.031) (Supplementary Table 2), indicating that the presence of this genotype is related to an advanced stage of the tumor, as also observed previously (37). However, we did not find an association between the presence of the most frequent MICA-sequence alleles (^*^002, ^*^008 and ^*^009/049) and tumor size or differentiation grade (Supplementary Table 2).

### Survival of GC Patients Based on MICA Alleles

**Figure 1:**
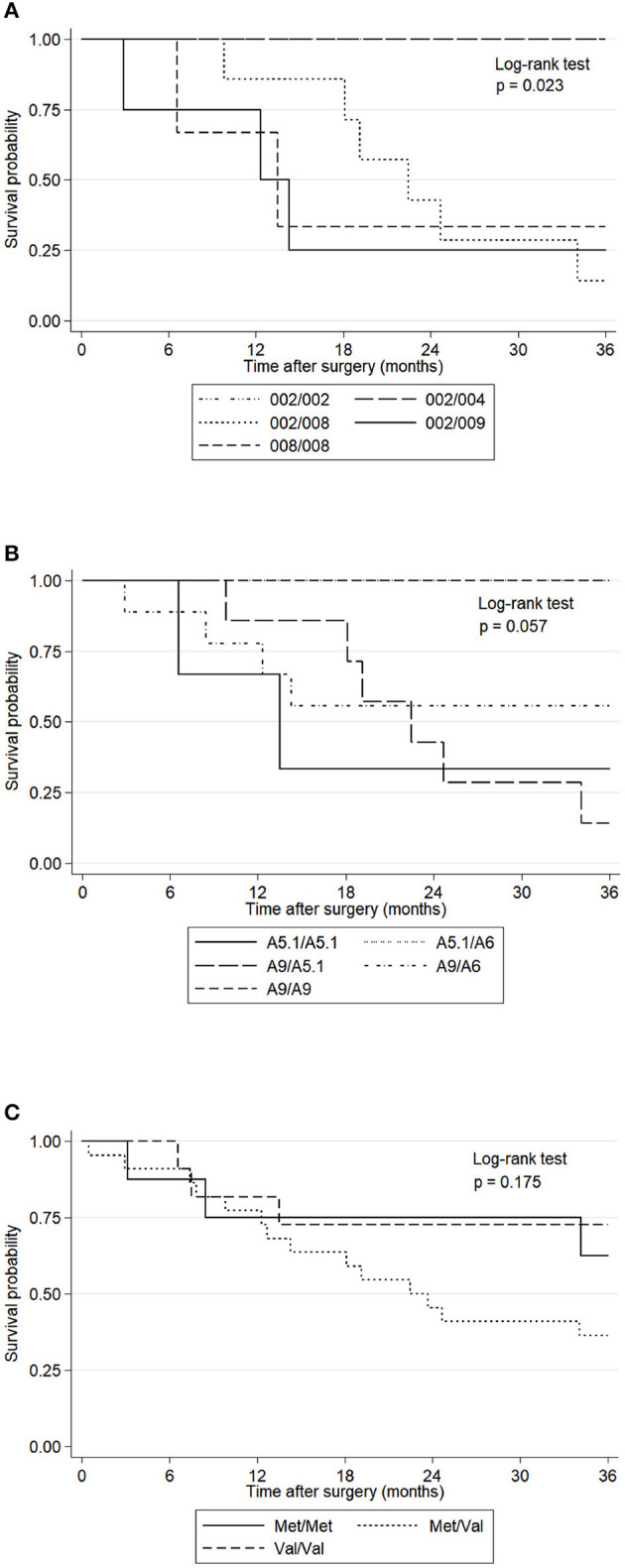
Kaplan-Meier curves for overall survival of GC patients with tumors at III and IV TNM staging, according to MICA-sequence, MICA-STR and MICA-129 genotype. The *p*-values were calculated by the Log-rank test. **(A)** The survival probability of GC patients with MICA*002/002 (*n* = 4) and MICA*002/004 (*n* = 4) alleles were significantly higher than that of MICA* 002/008 (*n* = 7) (*p* = 0.014) and MICA*002/009 (MICA*002/049) alleles (*n* = 4) (*p* = 0.040). The comparison between MICA*002/002 or MICA*002/004 and MICA*008/008 (*n* = 3) did not show significance (*p* = 0.070). **(B)** The survival probability of GC patients with MICA*A9/A9 (*n* = 4), MICA*A9/A5.1 (*n* = 7), MICA*A9/A6 (*n* = 9), MICA*A5.1/A6 (*n* = 3) and MICA*A5.1/A5.1 (*n* = 3) did not show significant differences (*p* = 0.057). **(C)** The survival probability of GC patients with the Met/Met genotype (*n* = 8), Met/Val (*n* = 22) and Val/Val (*n* = 11) did not show statistical differences (*p* = 0.175).

We analyzed the overall survival of GC patients according to MICA alleles during 36 months after gastrectomy. We excluded the patients with a TNM stating I and II to avoid conflicting interpretations due to the possibility of a higher survival in patients with a lesser advanced disease. First, we classified the patients based on the main alleles found among them, which included patients homozygous for MICA^*^002 or MICA^*^008 and heterozygous for MICA^*^002/009 (MICA^*^002/049), MICA^*^002/004, or MICA^*^002/008. The Kaplan-Meier curves and Log-rank test showed significant differences among these groups of patients. MICA ^*^002 and MICA^*^002/004 patients showed a higher survival rate than MICA^*^002/008 patients (*p* = 0.014) or MICA^*^002/009 (MICA^*^002/049) (*p* = 0.040). The survival distributions of homozygous patients for MICA^*^002 or heterozygous patients for MICA^*^002/004 were similar to that of homozygous patients for MICA^*^008 (*p* = 0.070). Likewise, no differences in the survival distributions of patients with MICA^*^002/008 compared to MICA^*^002/009 (MICA^*^002/049) or MICA^*^008/008 alleles could be detected (*p* = 0.611 and *p* = 0.909, respectively), neither between patients with MICA^*^002/009 (MICA^*^002/049) and those homozygous for MICA^*^008 (*p* = 0.883) (Figure 1A).

We also compared the survival curves of GC patients based on MICA-STR alleles (Figure 1B) and MICA-129 genotype (Figure 1C), which did not reach statistical significance (*p* = 0.057 and *p* = 0.175, respectively). Therefore, the MICA sequence, and not MICA-STR, may have a prognostic value in this type of cancer.

### Molecular Analysis of MICA-181 Residue

**Figure 2:**
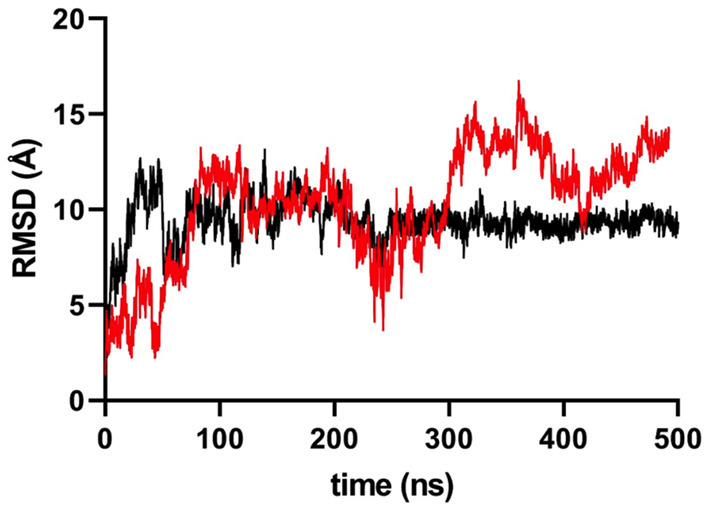
RMSD variation in MICA variant simulations. Black colored line represents MICA*004 (Arg181 variant), while red colored line represents MICA*009/049 (Thr181 variant). After 200 ns, MICA*004 remains constant, but MICA*009/049 displayed a less restrictive movement.

Since we found that the MICA-181 residue showed differences between GC patients and controls (Table 3), and the fact that this residue defines the MICA^*^009/049 (Thr181) and MICA^*^004 (Arg181) alleles, we decided to perform a molecular analysis of these proteins to evaluate the impact of this amino-acid residue in the dynamic properties of MICA. Molecular dynamics simulations of both proteins displayed a different behavior during the simulation time of 500 ns. MICA^*^009/049 displayed greater mobility compared to MICA^*^004, which revealed a more restricted movement at the end of the simulation time, during which the change in the root mean square deviation (RMSD) values remained almost constant, as observed in Figure 2.

**Figure 3:**
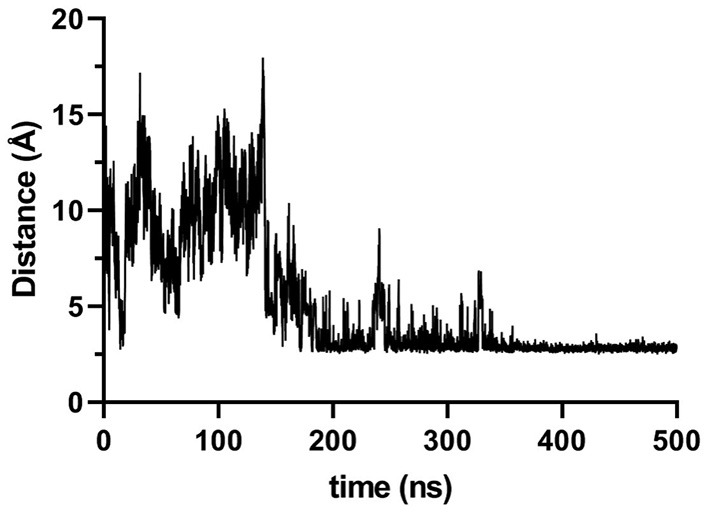
Distance, in Ångström, between Arg181 and Asp29 during simulation time of MICA. Distance was measured considering Nε of Arg181 and oxygen atoms of Asp29. The figure reveals that around 200 ns this hydrogen bond remains stable for the rest of the simulation.

**Figure 4:**
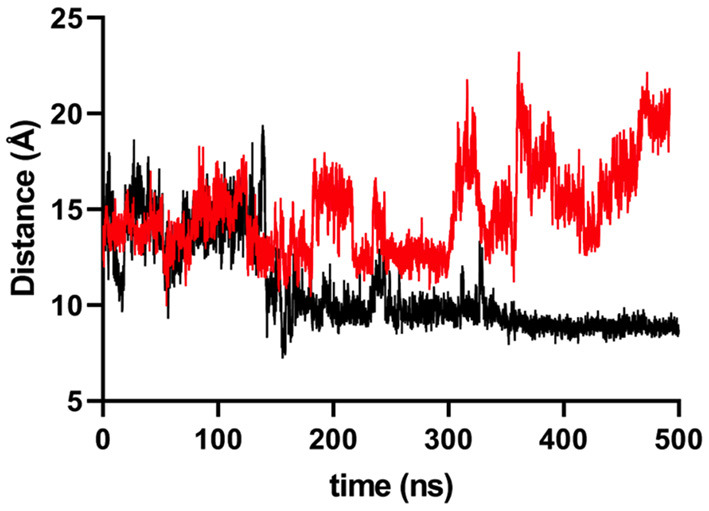
Distance, in Ångström, between Cα carbons of Thr181/Arg181 residues and Cα of Asp29 on MICA. Black colored line represents Arg181, while red colored line represents Thr181. Around 200 ns, the behavior of alleles differs due the hydrogen bond formation by the Arg181 and the Asp29.

During the simulation, we noticed that the Arg181 residue established an electrostatic interaction with Asp29 (Figure 3), which, according to the simulations, is responsible for the flattening of the curve for MICA^*^004. Based on this observation, it was possible to measure a distance of Cα carbons of both residues (Arg181 vs. Thr181) with Asp29 in order to compare the importance of this interaction. We observed a higher distance for Thr181 than Arg181 around 300 ns (Figure 4). This implies that Thr181 does not accomplish a stabilizing interaction, probably due to its shorter side chain, as compared to Arg181. This confirmed that there is a greater movement in MICA^*^009/049, as Thr181 does not display the electrostatic interaction with Asp29.

## Discussion

MICA, one of the main ligands to NKG2D receptor, has been considered an immunological target due to its participation in immune evasion mechanisms in cancer (38), including GC (5). MICA gene is highly polymorphic and, thus, originates different alleles based on its full-length sequence or STR (microsatellites) (MICA-STR). In this work, we identified MICA^*^002 (^*^A9) allele as the most frequent MICA variant in our cohort of GC patients and healthy controls, followed by MICA^*^008 (^*^A5.1) allele. In our study, both, patients as healthy controls shared the same geographic location; hence, the allelic frequencies described here are matched between cohorts. In this sense, the allele distribution found in our population differs from those reported in European, Asiatic, North American and Brazilian population (http://www.allelefrequencies.net), where the most frequent allele is MICA^*^008, followed by MICA^*^002 (39–43).

This difference could be explained by the ethnic diversity, which was not evaluated in the present work, and would certainly be recommended to be analyzed in future studies. Preliminary analysis of the rs67841474 in *MICA* gene, which corresponds to an indel that originates a frameshift variant (MICA^*^A5.1), has revealed that, in Chilean individuals from different ethnic origins, MICA^*^A5.1 is present in 20% of indigenous people from the North of Chile (Aymaras) in comparison to about 80% of indigenous people from the South of Chile (Mapuches) (44) (data available at http://genoma.med.uchile.cl:81/chilegenomico/). However, it is important to take into account that our cohorts are small when considering the variation that is exhibited by individuals in terms of MICA alleles; moreover, the frequencies would also be different depending on the population studied.

Even though it is clearly necessary to increase the cohort of study population for future investigations on NKG2D ligands (NKG2DL) polymorphisms, this time considering ethnical information and including human leukocyte antigen (HLA) analysis for a full characterization, we believe that our findings shed some light on the susceptibility and prognostic value of MICA polymorphisms in gastric cancer. Additionally, we also consider important to analyze the levels of soluble MICA in the serum of GC patients, which can be derived from the proteolytic shedding of MICA from the cell membrane, to evaluate whether these levels are related to MICA alleles, as some of the MICA variants could be more susceptible to this process. Such analysis would explain, at least in part, the broad interindividual variability of these levels in GC patients, which, in this particular type of cancer, appear to have a relatively low diagnostic impact (45); thus, MICA alleles could complement the diagnostic value of soluble MICA in this type of malignancy (46).

MICA polymorphic variants have been associated to several autoimmune and inflammatory diseases, as well as cancer (47, 48). Here, we observed that the presence of MICA^*^009/049 (MICA^*^A6) allele increases the susceptibility of developing GC, thus constituting a genetic risk factor for this type of cancer. In contrast, MICA^*^A5 allele was inversely associated with GC; therefore, this variant may be a protective factor for this disease, similar to findings previously reported for other types of cancer (49) and infectious diseases, such as tuberculosis (50).

MICA^*^009 allele has also been found in a high frequency in melanoma patients compared with controls (51). Additionally, both MICA^*^009 and MICA^*^004 (MICA^*^A6) have been associated with Behcet disease (18, 52–56) and rheumatoid arthritis (57). However, this variant was less frequent in patients with colorectal cancer (58), indicating that MICA alleles play a different role depending on the type of cancer, possibly due to cellular and molecular mechanisms that are particularly associated with different types of tumors. Accordingly, our results indicate a tendency for a higher frequency of MICA^*^004 among healthy individuals compared to GC patients. It is worth mentioning that MICA^*^009/049 and MICA^*^004 alleles produce a protein that differ at 181-residue, which makes it an interesting residue to analyze.

**Figure 5:**
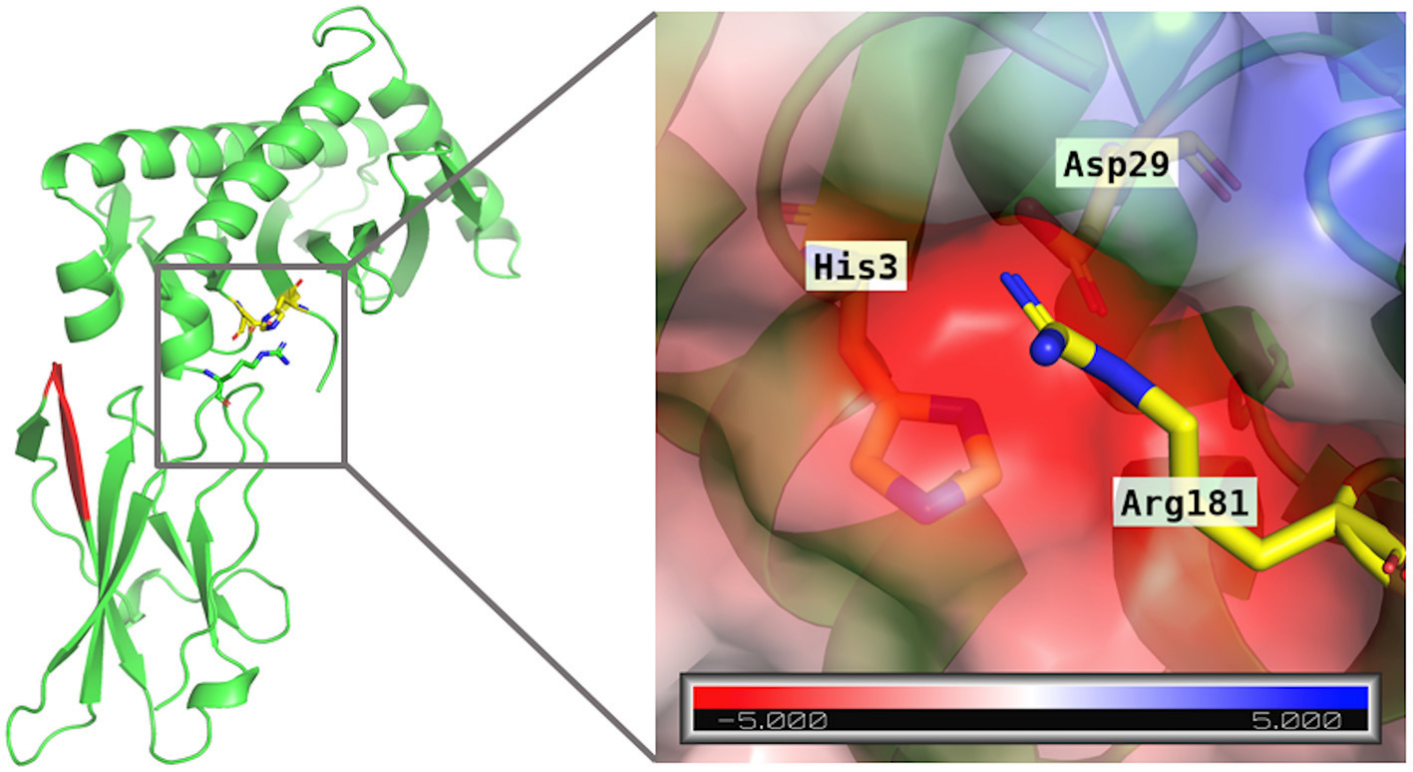
Electrostatic potential map for the crevice formed by residues His3 and Asp29 (yellow sticks) on MICA. The electrostatic potential map is given in kT/e units.

Based on the RMSD values and distance graphs described above, we hypothesize that different interactions between residues are achieved when both soluble MICA variants are simulated in a droplet of water. These results suggest that the interactions of MICA^*^004 are related to the physicochemical nature of Arg181, which bears a positive charge in its scaffold. This residue forms hydrogen bonds with neighboring residues, especially charged ones, as is the case of Asp29 in the simulation. Even though Thr181 residue can also form hydrogen bonds, this is a shorter and uncharged residue. A closer inspection of the interactions occurring for Arg181 reveals that not only is a coulombic interaction with Asp29 reinforced by hydrogen bonds, but it also displays a cation-π interaction with a terminal histidine residue (His3) at 4.5 Å. These interactions seem to be strong enough to keep the Arg181 stack in a crevice formed by these polar residues. In the case of Thr181 in MICA^*^009/049, these interactions are not observed during the simulation time, resulting in a more freely movement compared to Arg181. This observation implies that the change in only one amino acid can render a completely different dynamic behavior for both proteins. Figure 5 displays the Arg181 in the crevice formed by Asp29 and His3, which, in turn, displays a negative electrostatic potential surface, where Arg181 remains occluded. This is not observed with Thr181, which is not able to visit this site.

The MICA^*^009-HLA B^*^50, B^*^51 or B^*^52 haplotypes have been described in previous studies (59–61). Accordingly, HLA B^*^51 was found to be more frequent in *Helicobacter pylori*-positive pediatric patients with active gastritis and duodenal ulcer (62) and the HLA B^*^52 antigen has shown to be associated with lymph node metastasis in gastric cancer (63). Therefore, both HLA-B alleles and MICA^*^009/049 could to be factor risk in gastric cancer, so we suggest to consider this information for future studies.

When we analyzed the clinical characteristics of GC patients in relation to MICA polymorphisms, we observed that MICA-129, MICA-181 and MICA-sequence alleles were not related to tumor size, TNM stating or tumor differentiation grade in this type of cancer, whereas, tumors from MICA^*^A9/A6 heterozygote patients, who thus possess combinations of MICA^*^002 and MICA^*^009/049 or MICA^*^004, showed poorly differentiated tumors. These observations suggest that the transmembrane region of MICA may play a relevant role in GC progress. We suspect that the length of MICA transmembrane region, relative to the presence of six or more alanine residues, could implicate an increased susceptibility for proteolytic shedding of MICA by metalloproteases in the tumor microenvironment. If this is the case, the soluble levels of MICA would increase, which could negatively modulate the NKG2D receptor, favoring immune evasion mechanisms.

Here, we have also analyzed whether MICA variants affect the prognosis of GC. Our results showed that GC patients carrying the MICA^*^002 allele have better survival rates compared to GC patients carrying other MICA alleles. It has been previously demonstrated that the SNP rs9266825, which is part of MICA^*^002, ^*^007, ^*^018, ^*^017, ^*^001 alleles, is associated with increased survival rates in non-small cell lung cancer patients (64). Nevertheless, our study demonstrates, for the first time, the overall survival rates of GC patients based on the whole sequence of MICA gene.

We propose that certain MICA alleles have the potential to be more expressed on gastric tumor cells, depending on their cellular microenvironment, which may favor a better or worse immune response mediated by NK cells. According to our data, MICA^*^002 and MICA^*^004 alleles could be highly expressed on the surface of gastric tumor cells, which would trigger NKG2D receptor activation and an effective NK cell response. This is supported by other studies, which indicate that high-cell surface MICA/B expression in cancers of the digestive tract was associated with increased patient survival (65). In contrast, the levels of other protein variants, including MICA^*^009 and MICA^*^008, may be lower on tumor cell surface due to their shedding by metalloproteinases or release into the tumor milieu in extracellular vesicles, reducing target cell interaction with NK cells through the NKG2D receptor, thus affecting NK cell-mediated cytotoxicity. Support for this hypothesis comes from reports showing that patients with gastric tumors with high MICA expression had higher overall survival and disease-free-survival than patients bearing tumors with low MICA expression (66).

Our findings indicate that certain MICA alleles could have different effects on clinicopathological features of gastric tumor, such as the differentiation grade of tumor, as well as on patient overall survival after potentially curative gastrectomy, which may depend on the tumor microenvironment and regulation of MICA protein expression. In this sense, it will be interesting to analyze, in future studies, whether soluble MICA levels present any relationship with MICA alleles in GC patients.

In conclusion, MICA^*^009/049 allele increases the susceptibility to gastric cancer, whereas, MICA^*^A5 allele has a protective effect in this disease. MICA^*^002/002 and MICA^*^002/004 patients have higher survival rates than MICA^*^002/008 and MICA^*^002/009 (MICA^*^002/049) after surgery. Therefore, we consider that functional studies of MICA variants may help elucidate the mechanisms by which MICA confers protection or risk to GC development and prognosis, which may be a useful tool for the development of novel therapeutical approaches to treat this disease.

## Data Availability Statement

The raw data supporting the conclusions of this article will be made available by the authors, without undue reservation, to any qualified researcher.

## Ethics Statement

The studies involving human participants were reviewed and approved by Committee on Human Ethics Investigation of the Faculty of Medicine, University of Chile, and the Committee on Scientific Ethics of the Metropolitan Health Service of the Chilean Government. The patients/participants provided their written informed consent to participate in this study.

## Author Contributions

KT-S, CHR, PG-H, GZ-T, and MCM interpreted the data and wrote the manuscript. AC analyzed the data. RA interpreted the data. GZ-T, DM, KT-S, MM, VG, JR-S, ST, and MB performed the bioinformatics analysis and laboratory experiments. MCM supervised the work. All authors contributed to manuscript revision and approved the submitted version.

## Conflict of Interest

The authors declare that the research was conducted in the absence of any commercial or financial relationships that could be construed as a potential conflict of interest.
