# Supplementary material for: Major Histocompatibility Complex Class I-Related Chain A (MICA) Allelic Variants Associate With Susceptibility and Prognosis of Gastric Cancer
Source: Front Immunol. 2021 Mar 31;12:645528. doi: 10.3389/fimmu.2021.645528 (PMC8045969; doi:10.3389/fimmu.2021.645528)
Supplement: Supplementary file 1 [file Data_Sheet_1.doc]

Supplementary Material

| Table S1: MICA genotypes frequencies in GC patients and healthy controls | | | | |
| --- | --- | --- | --- | --- |
| MICA genotype | GC patients (n=44) | | Healthy Controls (n=50) | |
|  | n | (%) | n | (%) |
| 002/001 | 2 | (4.6%) | 0 | (0.0%) |
| 002/002 | 5 | (11.4%) | 3 | (6.0%) |
| 002/004 | 4 | (9.1%) | 7 | (14.0%) |
| 002/007 | 0 | (0.0%) | 2 | (4.0%) |
| 002/008 | 8 | (18.2%) | 10 | (20.0%) |
| 002/009 (002/049) | 4 | (9.1%) | 1 | (2.0%) |
| 002/010 | 1 | (2.3%) | 1 | (2.0%) |
| 002/011 | 2 | (4.6%) | 0 | (0.0%) |
| 002/017 | 0 | (0.0%) | 1 | (2.0%) |
| 002/019 | 1 | (2.3%) | 4 | (8.0%) |
| 004/004  004/010 | 0  0 | (0.0%)  (0.0%) | 1  1 | (2.0%)  (2.0%) |
| 004/019  008/001 | 0  1 | (0.0%)  (2.3%) | 1  0 | (2.0%)  (0.0%) |
| 008/004 | 2 | (4.6%) | 6 | (12.0%) |
| 008/007 | 1 | (2.3%) | 0 | (0.0%) |
| 008/008 | 3 | (6.8%) | 0 | (0.0%) |
| 008/009 (008/049) | 3 | (6.8%) | 0 | (0.0%) |
| 008/010 | 1 | (2.3%) | 1 | (2.0%) |
| 008/011 | 0 | (0.0%) | 2 | (4.0%) |
| 008/012 | 0 | (0.0%) | 1 | (2.0%) |
| 008/017 | 1 | (2.3%) | 0 | (0.0%) |
| 008/027 | 0 | (0.0%) | 1 | (2.0%) |
| 009/004 (049/004) | 1 | (2.3%) | 0 | (0.0%) |
| 009/007 (049/007) | 1 | (2.3%) | 1 | (2.0%) |
| 009/009 (049/049) | 1 | (2.3%) | 0 | (0.0%) |
| 009/010 (049/010) | 1 | (2.3%) | 1 | (2.0%) |
| 010/011 | 0 | (0.0%) | 2 | (4.0%) |
| 010/017 | 1 | (2.3%) | 0 | (0.0%) |
| 010/099 | 0 | (0.0%) | 1 | (2.0%) |
| 011/017 | 0 | (0.0%) | 1 | (2.0%) |
| 019/012 | 0 | (0.0%) | 1 | (2.0%) |

Note: We identified the MICA-sequence alleles in 44 out of 50 GC patients.

| Table S2: Clinical features of MICA genotypes on gastric cancer progress | | | | | | | | | | | | | |
| --- | --- | --- | --- | --- | --- | --- | --- | --- | --- | --- | --- | --- | --- |
|  | **Tumor size** | | | | | | **Differentiation grade of tumor ^(1)^** | | | | | | |
| MICA-129 polymorphism | Genotype | ≤ 5 cm | | > 5 cm | | p-value* | Well | | Moderate | | Poor | | p-valueⴕ |
|  | Met/Met | 2 | (11.1%) | 8 | (25,0%) | 0.212 | 0 | (0.0%) | 3 | (25.0%) | 7 | (23.3%) | 0.740 |
|  | Met/Val | 8 | (44.4%) | 18 | (56,3%) | 0.306 | 2 | (50.0%) | 6 | (50.0%) | 14 | (46.7%) | 1.000 |
|  | Val/Val | 8 | (44.4%) | 6 | (18,8%) | 0.055 | 2 | (50.0%) | 3 | (25.0%) | 9 | (30.0%) | 0.772 |
|  |  |  |  |  |  |  |  |  |  |  |  |  |  |
| MICA-181 polymorphism | Thr/Thr | 14 | (77.8%) | 28 | (87.5%) | 0.303 | 3 | (75.0%) | 11 | (91,7%) | 25 | (83.3%) | 0.556 |
|  | Thr/Arg | 4 | (22.2%) | 4 | (12.5%) | 0.303 | 1 | (25.0%) | 1 | (8.3%) | 5 | (16.7%) | 0.556 |
|  |  |  |  |  |  |  |  |  |  |  |  |  |  |
| MICA-Sequence | *008/008 + 008/X; where X represents a different allele that 009 (049) and 002 | 3 | (20.0%) | 6 | (20.7%) | 0.641 | 1 | (25.0%) | 2 | (20.0%) | 6 | (24.0%) | 1.000 |
|  | *002/002 + 002/X;  where X represents a different allele that 008 and 009 (049) | 4 | (26.7%) | 11 | (37.9%) | 0.345 | 1 | (25.0%) | 3 | (30.0%) | 9 | (36.0%) | 1.000 |
|  | *009/009 (049/049) +009/X (049/X); where X represents a different allele that *002 and *008 | 2 | (13.3%) | 3 | (10.3%) | 0.146 | 0 | (0.0%) | 0 | (0.0%) | 4 | (16.0%) | 0.556 |
|  | *002/008 | 3 | (20.0%) | 5 | (17.2%) | 0.562 | 1 | (25.0%) | 4 | (40.0%) | 2 | (8.0%) | 0.054 |
|  | *002/009 (002/049) | 1 | (6.7%) | 3 | (10.3%) | 0.488 | 0 | (0.0%) | 0 | (0.0%) | 3 | (12.0%) | 0.672 |
|  | *008/009 (008/049) | 2 | (13.3%) | 1 | (3.4%) | 0.050 | 1 | (25.0%) | 1 | (10.0%) | 1 | (4.0%) | 0.166 |
|  |  |  |  |  |  |  |  |  |  |  |  |  |  |
| MICA-STR ^(2)^ | *A5.1/A5.1 | 1 | (9.1%) | 2 | (10.0%) | 0.719 | 0 | (0.0%) | 1 | (10.0%) | 2 | (13.3%) | 1.000 |
|  | *A9/A9 | 2 | (18.2%) | 3 | (15.0%) | 0.595 | 0 | (0.0%) | 3 | (30.0%) | 2 | (13.3%) | 0.370 |
|  | *A9/A5.1 | 3 | (27.3%) | 5 | (25.0%) | 0.606 | 1 | (33.3%) | 4 | (40.0%) | 2 | (13.3%) | 0.297 |
|  | *A9/A6 | 2 | (18.2%) | 8 | (40.0%) | 0.202 | 1 | (33.3%) | 0 | (0.0%) | 7 | (46.7%) | **0.031** |
|  | *A5.1/A6 | 3 | (27.3%) | 2 | (10.0%) | 0.226 | 1 | (33.3%) | 2 | (20.0%) | 2 | (13.3%) | 0.792 |
| ⴕFisher’s exact test. Note: MICA-129, Met/Met alleles: MICA*002/002, *002/001 and *002/011; Met/Val alleles: MICA*002/008, *002/004, *002/009 (*002/049), *002/019,* 001/008, *017/010, *007/009 (007/049) and *007/008; Val/Val alleles: *008/008, *008/010, *008/004, *008/009 (*008/049), *009/010 (*049/010), *009/009 (*049/049) and *009/004 (*049/004). MICA-181 Thr/Arg alleles: MICA*002/004, *008/004 and *009/004 (*049/004). Thr/Thr alleles: All possible combinations different to Thr/Arg alleles. ^(1)^: We excluded 4 of 50 patients due to that the differentiation grade of tumor data was not available. ^(2)^: We included the most common MICA-STR alleles. We excluded the MICA*A4 and other alleles without STR sequence. | | | | | | | | | | | | | |

**(A)** **(B)**
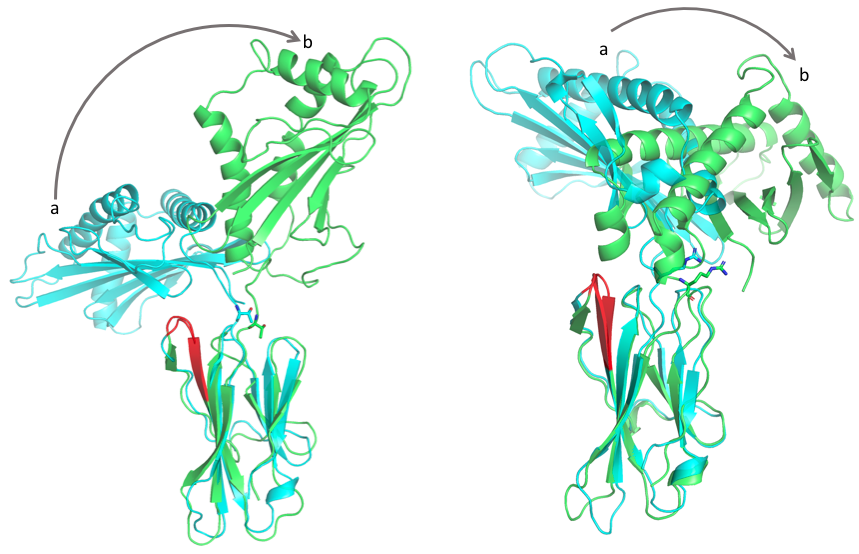


**Figure S1:** Ribbon diagrams of the MICA protein structure and its movements. **(A)** MICA*009/049 movement, where a represents the molecule initial position (light blue color) and b the final position (green color). **(B)** MICA*004 movement, characterized as a more restrictive movement due to the interaction of Arg181 with Asp29. Red color indicates the binding site of the Erp5 isomerase.
